# 

**Published:** 1899-04-22

**Authors:** 


*THE HOSPITAL.* "The standard of highest purity."—*Lancet.* April 22nd, 1899.

**CADBURY'S COCOA**
ABSOLUTELY PURE.

**CADBURY'S COCOA**

NO DRUGS OR ALKALIES USED.

### THE HOSPITAL

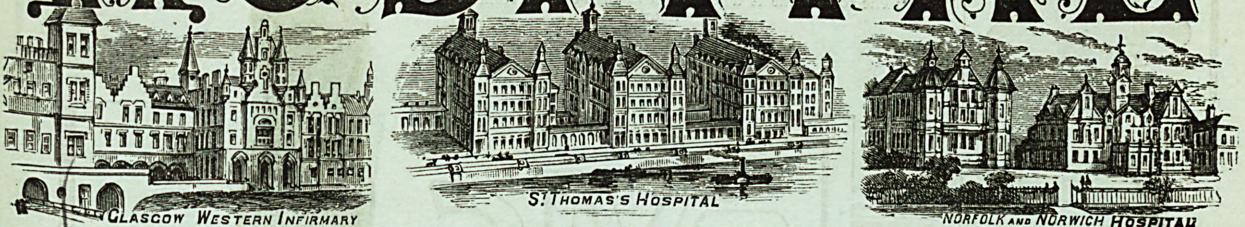

No. 656. Vol. XXVI. [Registered as a Newspaper.] Saturday, April 22nd, 1899. [Subscription Terms, see p. 58.] PRICE TWOPENCE.

